# Post-Processing Techniques for the Improvement of Liposome Stability

**DOI:** 10.3390/pharmaceutics13071023

**Published:** 2021-07-05

**Authors:** Ji Young Yu, Piyanan Chuesiang, Gye Hwa Shin, Hyun Jin Park

**Affiliations:** 1 Department of Biotechnology, College of Life Science and Biotechnology, Korea University, Anam-dong, Seongbuk-gu, Seoul 02841, Korea; 2018020235@korea.ac.kr; 2 Department of Food and Nutrition, Kunsan National University, Kunsan 54150, Korea; c.piyanan@hotmail.com

**Keywords:** post-processing techniques, liposome stability, freeze drying, spray drying, spray freeze drying

## Abstract

Liposomes have been utilized as a drug delivery system to increase the bioavailability of drugs and to control the rate of drug release at the target site of action. However, the occurrence of self-aggregation, coalescence, flocculation and the precipitation of aqueous liposomes during formulation or storage can cause degradation of the vesicle structure, leading to the decomposition of liposomes. To increase the stability of liposomes, post-processing techniques have been applied as an additional process to liposomes after formulation to remove water and generate dry liposome particles with a higher stability and greater accessibility for drug administration in comparison with aqueous liposomes. This review covers the effect of these techniques including freeze drying, spray drying and spray freeze drying on the stability, physicochemical properties and drug encapsulation efficiency of dry liposomes. The parameters affecting the properties of liposomes during the drying process are also highlighted in this review. In addition, the impact of using a protective agent to overcome such limitations of each process is thoroughly discussed through various studies.

## 1. Introduction

Drugs and bioactive compounds have an enormous potential for curing diseases, relieving pain, preventing disease or maintaining health. However, the administration of drugs or bioactive compounds through oral, topical, parenteral, rectal and nasal routes could be problematic due to unpredictable drug absorption and drug bioavailability at the site of action [1]. In order to overcome these problems, the application of drug delivery systems, which are well known as the method or process of transporting drugs or bioactive compounds to achieve the medicinal effects in humans, is very attractive especially in the pharmaceutical field of study [1,2]. Currently, many kinds of drug delivery systems have been developed by the encapsulation of drugs or bioactive compounds in various carriers such as liposomes [3,4], nanoemulsions [5], nanostructured lipid carriers [6,7] and micelles [8], aiming to increase drug bioavailability, control the drug release rate at the target organ and improve the safety and stability of the delivery systems themselves [1,9,10]. Among these carriers, liposomes, which are long circulating macromolecular carriers, have become the most interesting carriers because of their potential to deliver both hydrophilic and hydrophobic substances to cell membranes [2,11]. Even though the problems of stability and the physicochemical properties of conventional liposomes have been reported, the modification of liposomes by using biopolymers or the application of a few drying techniques to liposomes after formation have emerged as effective approaches to improve the stability and physicochemical properties of liposomes [1,11,12]. In comparison with the biopolymer modification of liposomes, which has been summarized in a previous study [11], the literature reviews related to the production of dried liposomes using drying techniques are limited. Therefore, this review paper aims to summarize the impact of commonly-used drying techniques such as freeze drying, spray drying and spray freeze drying on the stability and functionality of liposomes. The parameters affecting the properties of liposomes after drying and the current studies on the application of post-processing techniques to drug-encapsulated liposomes are also discussed.

## 2. Liposome Formulation

Liposomes are amphiphilic vesicular structures composed of an internal aqueous phase surrounded by one or several bilayer membranes of phospholipids. This amphiphilic nature allows liposomes to encapsulate both hydrophilic and hydrophobic substances into their structures without any association with surfactants [4]. The major constituents of liposomes—in general, phosphatidylcholine and cholesterol—do not convey surfactant characteristics, making liposomes the safest and most suitable drug delivery system for humans because of their biocompatibility, biodegradability and low toxicity [9,10]. The ability of liposomes to encapsulate unstable compounds such as drugs, vitamins, antioxidants and antibacterial agents has been reported to have numerous advantages such as a high encapsulation efficiency, high solubility and high protection capability with an increased bioavailability of active compounds. Those advantages also ultimately increase the ability of liposomes as delivery systems with an effective control-release feature at the site of action [9,13,14,15].

Liposomes can be manufactured by various methods such as thin film hydration techniques [16,17], ethanol injections [15,18], freeze drying [19,20], spray drying [21], microfluidization [22,23], heating [24], high shear dispersion [25], sonication [26], a superficial method [27,28] and the Mozafari method [29,30]. These methods can be used to prepare liposomes based on parameters such as the chemical and physical characteristics of active compounds, the average size, the polydispersity index, the stability of the prepared liposomes, the properties of liposome-dispersed medium and additional processes [31,32]. Recent studies on liposome preparation methods are summarized in Table 1.

As shown in Table 1, although general liposomes have several advantages as effective delivery systems, they may exhibit fatal disadvantages such as liposome self-aggregation, flocculation, coalescence and particle fusion, resulting in the gradual formation of larger vesicles and precipitation [33,34]. Liposomes can also be unstable due to temperature, pH and the presence of other components such as sugars and salts in a few food matrices [33]. Under these conditions, as the bilayer structure of liposomes loosens, the size increases due to aggregation and the stability of the liposome decreases. In addition, if the bilayer structure of the liposome is further widened, the encapsulated substances are lost. Therefore, the efficacy of liposomes as drug delivery system at the target point decreases. In severe cases, the bilayer structure of the liposome can be destroyed, resulting in a breakdown of safety. The encapsulated core material of liposomes can be leaked, degraded or decomposed due to the destabilization processes. As a result, the core material can deteriorate rapidly in the gastrointestinal tract and finally removed from the blood circulation [11,31,34,35]. Based on those reasons, the designed formation of liposomes is necessary to overcome the stability shortcomings of liposomes. Previous studies have reported the improvement of liposome stability by modifying the process of liposome preparation [11,36,37]. For instance, coating biopolymers on the surface of liposomes in order to adjust the particle size, particle morphology, surface charge, membrane fluidity and lipid chain ordering could increase the stability and time-circulation property of liposomes [11]. The incorporation of biopolymers into the structure of liposomes and the encapsulation of liposomes in hydrogels, films or nanofibers are such techniques that improve the stability and characteristic properties of liposomes [34,38]. However, even though the stability-enhancing effect of various kinds of biopolymer have been reported [11], critical challenges including the disruption of the liposome integrity, the agglomeration and coalescence of a biopolymer-coated liposome and the destabilization of the liposome bilayer may occur in the aqueous liposomes depending on the types of biopolymer and surrounding conditions [11].

Apart from the formation of biopolymer-associated liposomes, another promising way to improve the stability, bioactivity and bioavailability of liposomes is to perform additional processes, referred to as “post-processing techniques”, to the prepared aqueous liposome. In the next section of our review, the effectiveness of post-processing techniques on the stability and properties of dried liposomes are discussed.

## 3. Post-Processing Techniques for Liposomes

**Figure 1:**
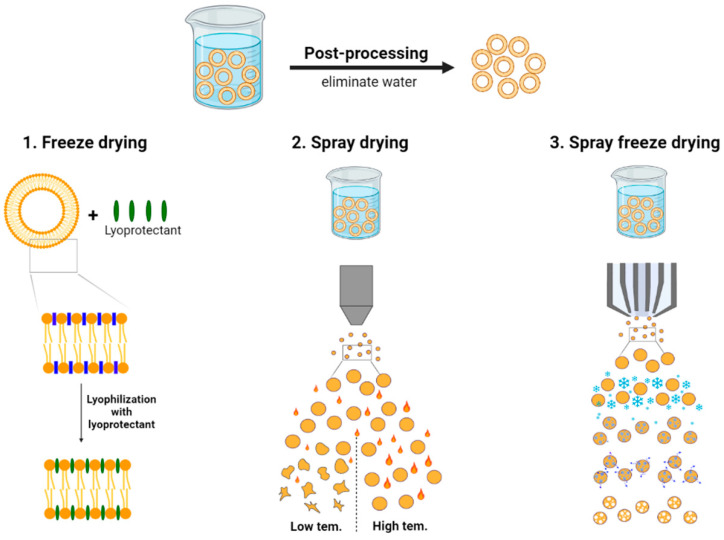
Schematic illustration of post-processing techniques.

The purpose of applying post-processing techniques to the prepared liposomes is, in general, to develop dried liposomes with a higher stability and a great accessibility for drug administration in comparison with aqueous liposomes [12]. In this section, the effects of common post-processing techniques including freeze drying, spray drying and spray freeze drying on the stability and bioavailability of liposomes are summarized according to the current studies (Figure 1). This review not only introduces the advantages and limitations but also discusses effective solutions to overcome the limitations of each drying technique.

### 3.1. Freeze Drying Process

Freeze drying or lyophilization is a water removal process that involves three major steps: 1. freezing (of a liposome-cryoprotectant mixture); 2. primary drying (where sublimation occurs) and 3. secondary drying (to reduce the residual moisture content) [39]. This process is one of the most widely used techniques for increasing the long-term stability of liposomes and preventing the degradation of sensitive substances encapsulated in the structure of liposomes [12,40,41]. However, the elimination of water through the freeze drying process may cause several adverse effects on the liposome such as changes in the vesicle size, a loss of membrane integrity, a loss of the encapsulated substance and an alteration of the rheological properties. These phenomena are caused by the interaction between the water molecules and hydrophilic head groups of phospholipids to form liposome bilayers [14,40,42]. According to Lopez-Polo et al. [14], the effect of the freeze drying process was investigated based on the physicochemical properties and rheological properties of rutin-loaded liposomes made from soy phospholipids. Even though the results in this study showed an increase in the storage stability of lyophilized rutin-loaded liposomes as well as the antioxidant capacity of rutin in comparison with those of non-lyophilized ones, a significant increase in the diameter and changes in the rheological parameters of liposomes caused by lyophilization were also reported.

Parameters such as the rate of freezing, liposome preparation method, bilayer composition and residual moisture content have been reported as critical factors involved in the stability and physicochemical properties of lyophilized liposomes [43]. For example, Franzé et al. [3] described that a rapid freezing rate, which involves the formation of small ice crystals, can reduce the disruption of the bilayer structure of liposomes. However, a slow freezing rate can have a negative effect on altering the liposome structure. A few studies have reported damage to the lipid bilayer due to the formation of large ice crystals as a result of the slow freezing process but a few studies, in contrast, reported the recovery of the bilayer membrane from deformations caused by mechanical stresses and osmotic pressure, which occurred due to less formation of ice crystals as a result of the slow diffusion of molecules across the bilayer when the external phase became concentrated due to freezing [3,41].

**Figure 2:**
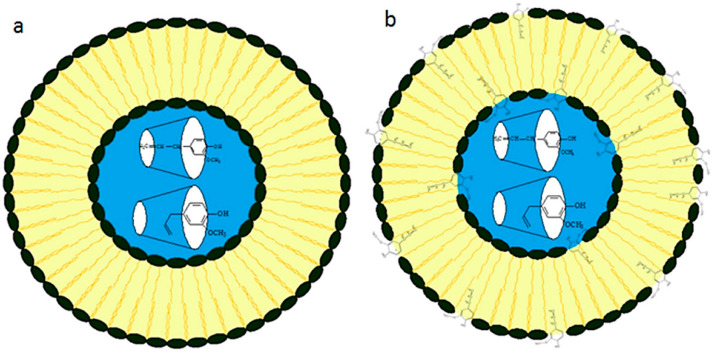
Suggested localization of eugenol (Eug) in Eug-loaded DCL (**a**) and DCL2 (**b**). Adapted with permission from ref. [44]. 2021, Elsevier.

Concerning the effect of the preparation technique on the stability of liposomes, Sebaaly et al. [44] prepared liposomes with 2-hydroxypropyl-β-cyclodextrin (HP-β-CD) and encapsulated eugenol as a core active material. The effect of the preparation methods, namely, (1) drug in cyclodextrin in liposomes (DCL) and (2) drug in cyclodextrin in liposomes prepared by a double loading technique (DCL2) (Figure 2), on the stability of lyophilized liposomes was then determined after a reconstruction in ultrapure water. In this study, the results showed that while DCL could maintain the stability of lyophilized liposomes, an increase in the average size and polydispersity index (PDI) as well as a loss of vesicle structures were observed in the lyophilized liposomes prepared by DLC2 after the reconstruction. According to the formulation of DCL2, where the drug was added to the organic phase whereas the inclusion complex was added to the aqueous phase of the liposome, this finding occurred because the presence of eugenol in the bilayer could interfere with the interaction between β-CD and the liposome; hence, leading to the rearrangement of the liposome structure [44].

**Figure 3:**
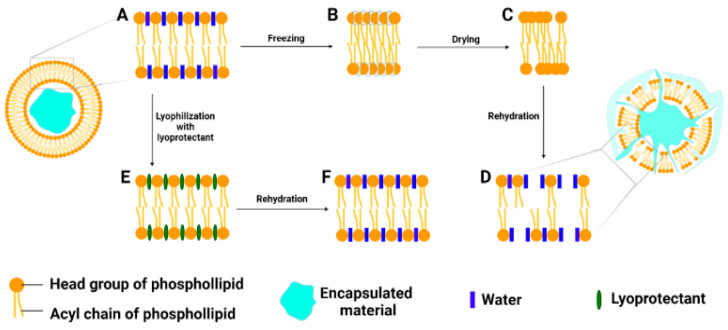
Mechanism of water replacement during lyophilization and rehydration. Adapted from [42], Elsevier, 2021.

In order to overcome the negative phenomena that occur during the freeze drying process of liposomes, adding a lyoprotectant to the liposome formulation has been extensively reported to inhibit vesicle aggregation, prevent the leakage of the encapsulated material and protect the phospholipid membrane from damage caused by ice crystals [41,42]. A variety of carbohydrates (monosaccharides, disaccharides, polysaccharides and synthetic saccharides), proteins (amino acids) and several alcohols that can interact with phospholipids could act as lyoprotectants (Table 2). To date, two lyoprotective mechanism-based disaccharides, the water replacement model and the vitrification model, have been proposed in the field of freeze-dried liposomes [41,42,45]. As shown in Figure 3, the former model explained the ability of sugar to replace water by interacting with phospholipids thereby maintaining the space between the phospholipid head groups (loose state) during freezing and reducing the van der Waals forces among the acyl chains of the phospholipids during drying. It is believed that the decrease in the phase transition temperature (Tm) of the bilayer as a result of the decrease in the van der Waals forces contributes to an increase in the stability and encapsulation efficiency of dry liposomes [42]. This mechanism is triggered by the presence of a lyoprotectant. However, the mechanism does not differ depending on the type of lyoprotectant or the glass transition temperature. The latter model describes the formation of a stable sugar glass matrix that can capture dry liposomes during freeze drying [41,45]. In this model, the presence of cryoprotectants between the bilayers, the glass matrix, with a high viscosity but low mobility, is believed to reduce the contact of membranes in proximity thus preventing the aggregation and damage of liposomes caused by ice crystals [41,45].

Currently, Toniazzo et al. [46] revealed that the use of sucrose as a lyoprotectant could maintain the average hydrodynamic diameter and zeta potential of lyophilized quercetin-loaded liposomes. The concentration of quercetin in the lyophilized liposomes also did not change significantly during storage for 100 days. In addition, the liposomes exhibited a low hygroscopicity and a low propensity for water adsorption. These results confirmed the efficacy of sucrose in protecting the lipid membrane and prolonging the stability of the dried liposomes.

Although lyoprotectants offer several benefits for the stability of lyophilized liposomes, it should be noted that the use of lyoprotectants always requires the optimal set temperature for lyophilization because liposomes are naturally sensitive to stress caused by changes in temperature and pressure. Depending on the type of lyoprotectant, a suitable freeze drying temperature should be optimized to maintain the bilayer integrity and avoid the collapse of the liposome structure [35]. To this end, consideration of the collapse temperature (T_c_) and the glass transition temperature (T_g_’) of the freeze-concentrated solution, which refers to the temperature at which visible or full collapse occurs and the temperature at which the heat capacity of the freeze-concentrated solution changes significantly, respectively, is necessary [47]. The optimal set temperature for the primary drying process can be lower than T_c_ but higher than T_g_’ [47]. To gain an insight into the collapse temperature of the liposome lyoprotectant sample during the freeze drying process, the collapse of ovalbumin-loaded liposomes influenced by sucrose was observed in real time by Hussain et al. [35] using a freeze drying microscope (FDM). In this study, the liposome sample was frozen at −50 °C at a rate of 10 °C/min. The morphology alteration of the ovalbumin-loaded liposome by adding a sucrose sample was performed when the sample temperature reached freezing point (−18.6 °C). In this study, the collapse of the sample was observed during the primary drying process when the sample temperature rose to −34 °C under a 0.1 mBar vacuum [35]. The optimal freeze drying condition for ovalbumin-loaded liposomes and the sucrose mixture was selected at 4 °C to −45 °C for freezing, −30 °C for primary drying and 30 °C for secondary drying (under a vacuum of 0.1 mBar), respectively.

### 3.2. Spray Drying Process

Owing to the disadvantages of the freeze drying process such as being time-consuming and expensive [34], the spray drying process is the most often used method in drug delivery systems for converting aqueous materials into a dried and powdery form [12,48,49,50]. Compared with freeze drying, the spray drying process is faster, cheaper and more suitable for the production of defined particles [12]. However, the main issue in the spray drying of liposomes is the occurrence of liposome aggregation, which leads to an increase in the particle size distribution and a leakage of the loaded active material during storage. To limit this aggregation, biopolymers with opposite charges such as proteins and carbohydrates have been utilized as protectants through spray drying to improve the kinetics and mechanical stability of liposomes [51,52]. Spray drying parameters including temperature, biopolymer type, biopolymer amount and spray gas flow affect the characteristics and morphology of the final powder [53,54,55]. The temperature of spray drying is related to the evaporation rate of moisture, the higher temperature and the faster evaporation rate. The temperature, in addition, also affects the state of the vacuole (a vapor bubble) formed with the particles. Under high temperature conditions (inlet/outlet temperature: 200 °C/125 °C), the vapor pressure in the vacuole rises and expands, leading to the formation of vacuoles within the particles. As the moisture is evaporated very quickly at the high temperature of spray drying, the surface of the particles becomes dry and hard. Therefore, the particles do not shrink; the shape is maintained and the surface of the particles appears smooth. On the other hand, when the spray drying temperature is low (inlet/outlet temperature: 120 °C/80 °C), the vapor condenses within the vacuole causing the particles to contract. In addition, the surface area of the particles is moist and flexible due to the slow moisture evaporation. Therefore, as the temperature decreases further, the vacuole becomes hollow and contracts, forming a wrinkled shape on the surface [51,54,56]. As the state of the vacuoles changes depending on the spray drying conditions, post-processing should be performed under appropriate conditions depending on the biopolymer used. Considering the effect of the biopolymer type, Khatib et al. [53] found an increase in the drug encapsulation efficiency of liposomes (hydrogenated soy phosphatidylcholine and cholesterol; HSPC) containing ciprofloxacin nanocrystals when using sucrose and lactose as protectants in comparison with trehalose. In this case, the ability of sucrose and lactose to maintain the liposome integrity was reported to be associated with the interactions, mainly hydrogen bonds, between those disaccharides and HSPC, decreasing the number of water molecules in the liposomes and, thus, stabilizing the liposome bilayer during the spray drying process [53,57]. However, depending on the composition of the liposome, trehalose or other biopolymers could also be considered appropriate protectants to interact and stabilize liposomes during spray drying.

Regarding the effect of the amount of biopolymer, Khatib et al. [53] also found that only the intermediate level of sucrose (41% *w*/*w*) had an impact on the increase in the particle size of liposomes whereas an amount greater or less than that did not. As a number of factors such as particle-particle interactions, osmotic pressure and lipid properties affect the structural rearrangement of liposomes during spray drying, a possible mechanism of this phenomenon has been described based on the interaction between sucrose and liposomes during spray drying [53]. In short, the high amount of sucrose retains the particle size of liposomes by two incidences: (1) by reserving the spaces between the adjacent liposomal vesicles through the interaction with phospholipids and (2) by maintaining the osmotic pressure between the liposomal membranes during drying, subsequently decreasing the amount of sucrose and thereby decreasing the protection ability, increasing the interaction between phospholipids, allowing drug leakage and increasing the liposomal aggregation upon rehydration. Once the amount of sucrose is lower than a certain level, drug leakage causes a difference in the osmotic pressure between the inside and outside of the droplets, thereby reducing the particle size of the liposome upon rehydration as a result of water diffusion (diffuse-out).

The utilization of biopolymers with spray drying has been confirmed to enhance the stability of liposomes [52,54]. For instance, according to a study by Gómez-Estaca et al. [52], the protective effect of alginate was observed to improve the stability of a soy phosphatidylcholine liposome during spray drying and storage. The study by Akgün et al. [54] also revealed that spray drying of liposomes containing a sour cherry extract with maltodextrin could protect the liposome membrane thereby maintaining the loaded phenolic compounds and their antioxidant activity.

However, a few antagonistic results regarding the effect of spray drying with protectants on the encapsulation efficiency of liposomes have also been reported because the degradation of the membrane integrity and leakage of the loaded active compounds can be caused by heat and dehydration during spray drying [34]. In this respect, coating an active compound-loaded liposome with chitosan or sodium alginate prior to mixing with the protectant and spray drying has been revealed to be an effective approach to improve the rigidity of liposomal membranes against heat. Following the study by Sarabandi et al. [34], coating nanoliposomes (phosphatidylcholine) containing a flaxseed peptide with chitosan before spray drying with maltodextrin could preserve the encapsulation efficiency of nanoliposomes after spray drying (with maltodextrin) and storage when compared with that of an uncoated nanoliposome. As chitosan interacts with the polar head of phospholipids through electrostatic interactions [58], the pores on the surface of the nanoliposomal membranes are then covered by chitosan, which in turn retard the release of the loaded flaxseed peptide during storage and after rehydration. In addition, a chitosan coating preserved the antioxidant capacity of a flaxseed peptide during spray drying [34]. In a study by Altin et al. [51], the spray drying of chitosan-coated liposomes containing a phenolic extracted from cacao hull waste also enhanced the bioaccessibility of phenolic compounds extracted in terms of the total phenolic and total flavonoid contents after the in vitro digestion.

### 3.3. Spray Freeze Drying Process

**Figure 4:**
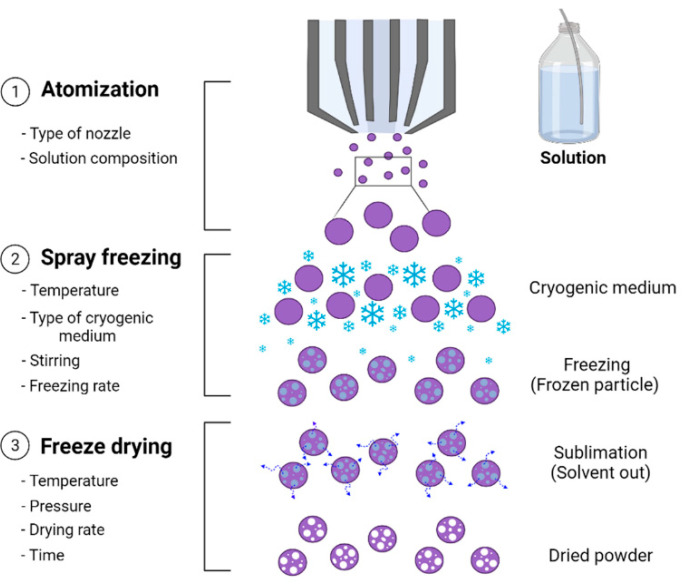
Illustration showing the steps involved in typical spray freeze drying. Adapted with permission from ref. [1]. 2021, Elsevier.

Freeze dying and spray drying processes have been widely used to improve the stability and encapsulation efficiency of liposomes. The advantages and disadvantages of each method are summarized in Table 3. The main advantage of the spray drying process is the production of defined and powdery forms of particles but loaded active compounds can be degraded owing to the impact of heat. Although the freeze drying process is suitable for preserving thermolabile substances, it is time consuming and, depending on the nature of the materials, the dry cake form of the final product can be very hygroscopic; hence, it may need to be kept in a specific container to prevent moisture sorption. To combine these advantages and compensate for the limitations of these methods, a spray freeze drying process was developed to provide dry products with a high stability [59]. The spray freeze drying process consists of three major steps: atomization, freezing and freeze drying [60]. As schematically illustrated in Figure 4, atomization is the first step in the conversion of the prepared feed solution into spherical droplets through an atomizer. Subsequently, the freezing and freeze drying steps are performed to solidify the droplets using a cryogenic agent (or cold fluid) and sublimate the ice at a low temperature and pressure, respectively [59]. The physicochemical properties of the final product after spray freeze drying such as the particle size, particle morphology, density and porosity have been found to vary depending on the type of atomizer, freezing time and the composition and concentration of the feed solution.

The type of atomizer has been reported to be the most important factor that determines the particle size and size distribution of the final product [1,59]. Depending on the expected particle size, various nozzles have been utilized such as a hydraulic nozzle (120–250 μm in size) and a two/four-fluid pneumatic nozzle (5–100 μm in size). In addition, ultrasonic nozzles and piezoelectric droplet-stream generator nozzles have also been used [60]. The spray freeze drying technique is considered to be a time-efficient technique when compared with the conventional freeze drying process where the spherical droplets generated from an atomizer have a large surface area and are therefore rapidly solidified by direct contact with the cryogenic agent during freezing. Unlike conventional freeze drying, the cryogenic agent used in the spray freeze drying process does not crystallize and the agent is removed in the sublimation step [60]; thus, phase separation does not occur in the final products. The freezing time relates to the types of cryogenic agents, which can be liquid nitrogen, liquid carbon dioxide and liquid argon [61]. Among these types of cryogenic agents, liquid or gas nitrogen is frequently used because of their inertness, density and low boiling point [60]. Liquid nitrogen provides a supercooling condition to the droplets and allows the formation of fine ice crystals [59], which are subsequently sublimated at low temperatures and pressures. After sublimation, a product with a high porosity characteristic is generated [62]. The solid content in the feed solution is also important for maintaining the particle morphology of the formed droplets. In this assertion, Vishali et al. [1] reported that an insufficient solid content in the feed solution could interrupt the spherical morphology of the droplets due to the mechanical instability of the individual particles.

**Figure 5:**
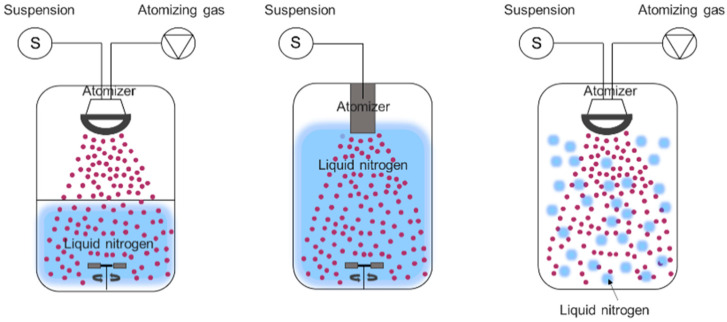
Illustration of different spray freezing techniques. Adapted from [60], MDPI, 2020.

The spray freeze drying process can be classified into three categories according to the physical state of the cryogen used in the spray freezing step. As shown in Figure 5, the three categories are classified as: (1) spray freezing into vapor over liquid (SFV/L), (2) spray freezing into liquid (SFL) and (3) spray freezing into vapor (SFV) [60]. The SFV/L technique (Figure 4) is one of the most commonly-used techniques for spray freeze drying of pharmaceutical substances. In this technique, the droplets obtained from an atomization step begin to freeze slowly once they pass through the cold vapor gap above the boiling point of the cryogenic liquid. The droplets are then completely frozen after coming into contact with the liquid cryogen [60].

The SFL technique (Figure 5) uses an insulated spray nozzle, which is directly connected to the cryogenic liquid. Hence, the droplets begin to freeze immediately upon contact with the cryogenic agent [60]. Lastly, the SFV technique provides the freezing of droplets when they come into contact with a gaseous cryogenic medium in the chamber (Figure 5).

**Figure 6:**
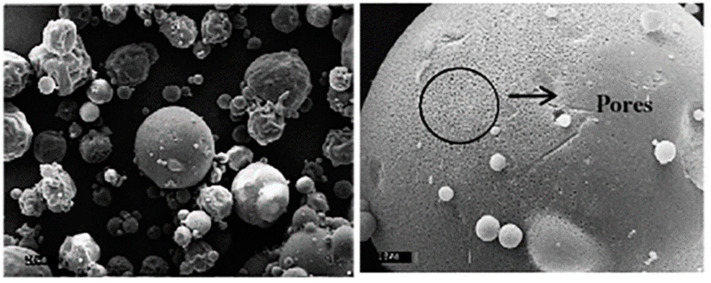
Porous sphere after the spray freeze drying process. The figures are reused with permission from Elsevier [62].

Compared with the conventional freeze drying method, the products from the spray freeze drying process are smaller and have a narrower size distribution owing to the rapid freezing and nucleation rate. Important characteristics such as spherical morphology, high porosity (Figure 6) and high solubility result in the final products being reconstituted without agglomeration after rehydration [62,63]. The rehydration and agglomeration results of freeze drying and spray freeze drying are different. This is because the freezing steps of the two methods are different. During the freezing step of freeze drying, it is divided into a phase containing ice and a phase containing a cryoprotectant. However, when they are frozen together, a thin crust is formed, which causes aggregation and slows the reconstitution of the product [64]. In addition, it interferes with the movement of the water vapor, resulting in the heating of the product and its fusion. On the other hand, spray freeze drying freezes into fine droplets, which freeze efficiently and quickly due to the large surface area. Rapid cooling causes thin interstitial regions. The presence of thin interstitial regions characterized by very small dendrite-shaped crystals results in a rapid reconstitution, the prevention of aggregation and improved stability in the freezing step of spray freeze drying [65,66].

The spray freeze drying process offers several advantages to the liposome particles as a drug delivery system. For example, powders dried through the spray freeze drying process can be stably and continuously released through various routes such as pulmonary and oral administration [1]. According to the study of Ye et al. [67], clarithromycin liposomal powder (CLA-Lips-DPIs) was successfully developed using an ultrasonic spray freeze drying technique. In this study, an ultrasonic nozzle was used to generate the spherical liposomal droplets, which were immediately frozen once in contact with liquid nitrogen. After the freeze drying step, the small (micron size) and highly porous liposomal particles were obtained. The effect of lyoprotectants (sucrose, trehalose and mannitol) on the particle size and morphology, moisture absorption, drug content uniformity, encapsulation efficiency and aerosolization performance of the clarithromycin liposomal powder were also investigated in this study [67]. The results found that the type and concentration of lyoprotectants affected the particle size and encapsulation efficiency of the dry liposomes. Liposomes formulated with sucrose (15%) contained the smallest particle size, the most uniform distribution and the highest encapsulation efficiency when compared with those formulated with trehalose and mannitol. However, liposomes formulated with sucrose also quickly absorbed moisture; therefore, this was not suitable for long-term stability. In order to improve the moisture protection ability, mannitol (15%) was added to the formulation due to its high crystallinity. Lyophilized liposome powder generated from the formulation of sucrose (5%) and mannitol (15%) was small, spherical and highly porous. The powder exhibited 92.14% and 0.09% of drug recovery and drug content uniformity, respectively. In addition, the powder also remained stable for at least three months with a high aerosol efficiency (emitted dose N, 85%; fine particle fraction 43–50%) [67]. Based on the scanning electron microscope (SEM) study, both the surface and inside structure of the dried powder containing 5% sucrose and 15% mannitol were filled with liposome particles. This result was reported to be associated with the breaking of the skeleton of the lyoprotectants into liposome-containing fragments, which increased the aerosol dispersion of the liposome during testing. Overall, these important characteristics including a small size, high porosity, high moisture protection ability, high drug recovery, high aerosolization performance and high stability indicated the suitability of that lyophilized powder as an effective drug delivery system for pulmonary administration (inhalation) [67].

According to Fukushige et al. [68], hyaluronic acid also exhibited a lyoprotective effect on the increasing stability and decreasing cytotoxicity of a liposome-protamine-DNA complex (LPD) encapsulated with small interfering RNA (siRNA) after spray freeze drying. Liposome particles approximately 30 μm in size were highly porous and highly stable because the change in their particle size was negligible after redispersion. This finding can be related to the suppression effect of hyaluronic acid on the aggregation of particles as a result of steric hindrance [68,69].

## 4. Conclusions

The destabilization of aqueous liposomes through self-aggregation, coalescence, flocculation and precipitation can shorten the shelf life and eventually lead to the decomposition of liposomes. The primary purpose of applying post-processing techniques such as freezing drying, spray drying and spray freeze drying to liposome formulations is to enhance the stability of the liposomes, which in turn increases the drug encapsulation efficiency of the liposomes. Freeze drying is suitable for liposomes composed of heat-sensitive substances but the stability of lyophilized liposomes can be lost depending on several factors such as the freezing rate, liposome preparation method, bilayer composition and residual moisture content. The optimization of these factors or the utilization of appropriate lyoprotectants has been shown to improve the stability and encapsulation efficiency of lyophilized liposomes. Owing to a few disadvantages of the freeze drying process, the spray drying technique is the most practical technique used for the production of defined liposome particles. Even though the agglomeration of dried and powdery liposomes after spray drying could result in an increase in the particle size and leakage of the loaded active material during storage, applying biopolymers with opposite charges (against liposomes) to the formulation of liposomes or coating liposomes with chitosan before spray drying has been confirmed to enhance the stability and maintain the vesicle structure of liposomes. The application of the spray freeze drying process to liposomes was also discussed in this review paper. The process in which the unique features of freeze drying and spray drying are combined resulted in final liposome particles with important properties such as spherical morphology, a high porosity and a high solubility. Spray freeze drying is mainly used in the pharmaceutical field at present but is increasingly being applied to the food industry. The delivery system was also started in the pharmaceutical field as a drug delivery system but now it is a technology that is widely used in the food industry as well. In pharmacy and food, research to increase the solubility of poorly soluble/insoluble substances that are not well soluble in water and to have a maximum efficiency in the body and ultimately to increase the stability of substances is being conducted in common. Therefore, spray freeze drying will also be used more often than now to improve the stability of liposomes in the future and it is predicted that it will be the primary technique.

## Figures and Tables

**Table 1:** Preparation techniques and recent studies of liposomes.

Liposome Formulation	Liposome Preparation Technique	Core Materials	Shell Materials	Protectant	Main Result	Ref.
**Liquid State**	**Thin Film Hydration** **(+ Extrusion/Sonication)**	Curcumin	Soybean lecithin, cholesterol	Chitosan	Improved stability of curcumin-loaded liposomes	[16]
		Fish hydrolyzed collagen	Soy phosphatidylcholine with cholesterol or glycerol	-	Enhanced bioactivities and stability of hydrolyzed collagen	[17]
	**Ethanol Injection**	Ethanolic coconut husk extract	Phosphatidylcholine, cholesterol	-	Enhanced antibacterial properties, improved dark color	[15]
		Cinnamaldehyde	Egg yolk lecithin, Tween 80	Chitosan	Increased encapsulation efficiency, antibacterial activity and storage stability	[18]
	**Microfluidization**	Branched-chain amino acids	Phosphatidylcholine, cholesterol, palmitic acid, hexadecylamine	Chitosan, pectin	Improved colloidal and intestinal stabilities of encapsulated branched-chain amino acids	[22]
		Green tea extract	Soybean lecithin	Gum arabic, Whey protein, lysozyme, chitosan	Increased storage stability of liposomes	[23]
	**Heating** **(+ Sonication)**	Rutin, glycerol, cellulose nanofibers	Soybean lecithin	HPMC	Improved appearance, increased apparent viscosity, decreased cohesive energy of the coating suspension	[24]
	**High Shear Disperser**	Black mulberry (*Morus nigra*) extract	Lecithin	Chitosan, maltodextrin	Protected anthocyanin content, enhanced in vitro bioaccessibility of anthocyanins	[25]
	**Sonication**	Shrimp oil	Phosphatidylcholine	-	Improved stability and nanoencapsulation efficiency, minimized fishy odor	[26]
	**Superficial**	Olive pomace extract	l-α-Phosphatidylcholine	-	Increased encapsulation efficiency of polyphenol compounds	[27]
		Limonene	l-α-Phosphatidylcholine	-	Increased encapsulation efficiency of limonene	[28]
	**Mozafari**	Green tea extract	Lecithin, glycerol	-	Improved stability and antioxidant activity of green tea extract	[29]
		Algal extract	Soybean lecithin	-	Increased stability, maintained the antioxidant activity of algal extract	[30]
**Solid State**	**Freeze Drying**	Calcein, 5-fluorouracil, flurbiprofen	Soybean phosphatidylcholine, cholesterol	Sucrose, lactose, mannitol	Increased encapsulation efficiency	[19]
		Glycyrrhetinic acid	Soybean phosphatidylcholine, cholesterol	Lactose, sucrose, trehalose, mannitol	Increased water solubility and encapsulation efficiency	[20]
	**Spray Drying**	Lopinavir	Phospholipon 85G^®^, cholesterol	-	Increased stability and % entrapment efficiency	[21]

**Table 2:** Classification of lyoprotectants.

1. Carbohydrate		2. Protein [39]	3. Polyol [39]
**1.1. Mono and disaccharides [39,41]**	**1.2. Oligo and polysaccharides [39]**	Glycine	Mannitol
Glucose (dextrose)	Raffinose	Gelatin	Sorbitol
Fructose	Hydroxypropyl-β-cyclodextrin (HP-β-CD)	Proline	Glycerol
Mannose	Chitosan	Glutamine	Ethylene glycol
Maltose	Maltodextrin	Betaine	Propylene glycol
Sucrose	Inulin	Arginine	Polyvinyl alcohol
Trehalose	Dextran	Lysine	
Cellobiose	Hyaluronan	Histidine	
Lactose			

**Table 3:** Advantages and disadvantages of three post-processing techniques.

Post-Processing Technique	Step	Advantages	Disadvantages
**1. Freeze Drying**	(1) Freezing (2) Primary drying (3) Second drying	Simple technique Suitable for thermolabile substances	Time-consuming Expensive Lyoprotectant required Aggregation often occurs
**2. Spray Drying**	(1) Atomization (2) Drying	Convenient and most used Time and cost-efficient Suitable for the production of defined particles	Delicate temperature control Biopolymers required Not suitable for thermolabile substances
**3. Spray Freeze Drying**	(1) Atomization (2) Freezing (3) Freeze drying	Time and cost-efficient No additives required Suitable for thermolabile substances High stability	Cryogenic required

## Data Availability

No new data were created or analyzed in this study. Data sharing is not applicable to this article.
